# Assessing the relationship between neural health measures and speech performance with simultaneous electric stimulation in cochlear implant listeners

**DOI:** 10.1371/journal.pone.0261295

**Published:** 2021-12-13

**Authors:** Florian Langner, Julie G. Arenberg, Andreas Büchner, Waldo Nogueira

**Affiliations:** 1 Department of Otorhinolaryngology, Hannover Medical School and Cluster of Excellence Hearing4all, Hanover, Germany; 2 Department of Otolaryngology, Massachusetts Eye and Ear, Harvard Medical School, Boston, MA, United States of America; University of California, Los Angeles, UNITED STATES

## Abstract

**Objectives:**

The relationship between electrode-nerve interface (ENI) estimates and inter-subject differences in speech performance with sequential and simultaneous channel stimulation in adult cochlear implant listeners were explored. We investigated the hypothesis that individuals with good ENIs would perform better with simultaneous compared to sequential channel stimulation speech processing strategies than those estimated to have poor ENIs.

**Methods:**

Fourteen postlingually deaf implanted cochlear implant users participated in the study. Speech understanding was assessed with a sentence test at signal-to-noise ratios that resulted in 50% performance for each user with the baseline strategy F120 Sequential. Two simultaneous stimulation strategies with either two (Paired) or three sets of virtual channels (Triplet) were tested at the same signal-to-noise ratio. ENI measures were estimated through: (I) voltage spread with electrical field imaging, (II) behavioral detection thresholds with focused stimulation, and (III) slope (IPG slope effect) and 50%-point differences (dB offset effect) of amplitude growth functions from electrically evoked compound action potentials with two interphase gaps.

**Results:**

A significant effect of strategy on speech understanding performance was found, with Triplets showing a trend towards worse speech understanding performance than sequential stimulation. Focused thresholds correlated positively with the difference required to reach most comfortable level (MCL) between Sequential and Triplet strategies, an indirect measure of channel interaction. A significant offset effect (difference in dB between 50%-point for higher eCAP growth function slopes with two IPGs) was observed. No significant correlation was observed between the slopes for the two IPGs tested. None of the measures used in this study correlated with the differences in speech understanding scores between strategies.

**Conclusions:**

The ENI measure based on behavioral focused thresholds could explain some of the difference in MCLs, but none of the ENI measures could explain the decrease in speech understanding with increasing pairs of simultaneously stimulated electrodes in processing strategies.

## 1. Introduction

Cochlear implants (CI) are the most successful sensory implant of our time and provide some restoration of hearing to those with severe to profound hearing loss [1]. CIs use sophisticated signal processing to transpose the acoustic signal into a pattern of electrical stimulation that activates the auditory nerve. Performance is highly variable among CI users and depends most prominently on the age at onset of severe to profound hearing loss [2, 3], duration of deafness [4–6] and experience with the CI [7, 8]. These demographics, the etiology and the physical attributes of the electrode array, which taken together, describe the electrode-neural interface (ENI). The quality of the ENI is defined by how effectively the electrodes of the CI activate the auditory neurons, the target of CI electrodes. ENI is influenced by neural health [number of healthy auditory nerve fibers, see 9], electrode position [10] and abnormal tissue/bone growth in the cochlea [11]. The following methods have been used to estimate ENI: detection thresholds with focused electrical fields [12–14], CT imaging data [12, 15, 16], psycho-physical tuning curves [15], intracochlear resistance estimated with electrical field imaging [14], polarity sensitivity [17–19] and electrically evoked compound action potential (eCAP) inter-phase gap (IPG) difference measurements [20, 21]. While audiologists have some flexibility to set parameters in the programming software of each manufacturer, the time to find the right strategy and setting is untenable in most clinical settings. Both programming adjustments and patient adaptation occur the most during the first year after implantation when speech perception scores improve rapidly [4]. It is desirable to find the most efficient way of stimulating the auditory nerve, which is why this study focuses on various measures to estimate the quality of the ENI. We then correlate those measures with speech perception performance data from stimulation strategies that vary in the amount of interaction among CI channels.

Sound coding strategies have implemented simultaneous stimulation of pairs of adjacent electrodes (virtual channels) to steer current to locations between physical electrode contacts, that reduces the peak current of the device, and consequently reduces the overall power consumption [22–26]. The current-steering strategy can be implemented sequentially, where each virtual channel is stimulated one at a time, referred to as ‘Sequential’, or simultaneously/in parallel, such that two virtual channels, spaced by 8 electrodes are stimulated simultaneously, referred to as ‘Paired’, thereby also increasing the rate at which electrodes can be stimulated [27]. Langner et al. [27] compared speech performance and spectral resolution measures with a Sequential strategy and two simultaneous strategies with two (Paired) and three (Triplet) pairs of channels stimulated simultaneously, concluding that the change in performance was highly individual, with no consistent differences observed between the Sequential and the Paired condition. However, on average CI listeners’ performance significantly decreased with the Triplet strategy. One likely explanation for this reduction in performance is increased channel interaction with Triplet due to the simultaneous stimulation, which was indicated in the significant effect of strategy on current difference (displayed as current savings in percent by Langner et al.). Langner et al. [28] also found large loudness summation effects when comparing single and dual-channel stimuli, further indicating that simultaneous stimulation increases the magnitude of channel interaction and the difference therein between strategies such as Sequential, Paired and Triplet. The hypotheses to be tested in the present study were that the ENI is influencing these channel interactions and that this relationship is reflected in the way CI users with a certain ENI perform with the presented strategies. Furthermore, this exploratory approach tries to answer the question whether individual loudness summation effects (by increasing the number of simultaneously stimulating channels) are related to the ENI and in turn may have an effect on speech understanding performance.

CI technology offers unique opportunities to characterize physical measures of the environment in the cochlea as well as physiological responses of the auditory nerve in individual listeners using reverse telemetry. This technology enables researchers and clinicians to collect these measures non-invasively. Recently, several investigators have attempted to characterize the ENI of CI users, correlating various speech performance measures to those thought to estimate the quality of the ENI [13, 15–18, 20, 29–35]. In the recent past, several studies investigated objective measures that correlated with histological data, suggested to be a good measure for characterizing the ENI. Most notably, Prado-Guitierrez et al. [36] used the auditory brainstem response and eCAP to examine the effect of stimuli with different pulse durations and interphase gaps (IPG) on neural survival in an animal model. Animals with better neural density had larger reductions in peak amplitudes and growth function slopes with increases in inter-phase gap than those with fewer neurons. Their results led to other studies of the IPG effect, here specified as IPG slope effect since this AGF aspect was used in the presented study, as a measure of neural density. A follow-up study was done by Ramekers et al. [34] who quantified the IPG slope effect and AGF in many ways. The authors found two interesting significant correlations: between the cell count and the difference of the slope of the AGFs with different IPGs, as well as between the cell count and the peak amplitude of the AGF. One recent study used the eCAP AGF characteristics in relation to speech performance in bilaterally implanted human CI users (Schvartz-Leyzac & Pfingst [37]) and they found correlations between the IPG slope effect (in their study the difference in slope when changing from 7 to 30 μs IPG) and the difference in speech performance between both ears/devices with sentences in speech-shaped noise and in quiet. While general performance across participants did not correlate with IPG slope, it did correspond with which ear a CI user was performing better with (with a large IPG slope effect indicating better performance in the form of lower speech reception threshold). Other studies also found evidence that the IPG slope effect may relate to neural density in humans [21, 33, 35]. The study introduced here attempts to relate AGF characteristics to the individual differences and the general outcome of speech performance with Sequential, Paired and Triplet speech processing strategies. We hypothesize that a large IPG slope effect, suggesting good neural health/high number of active auditory nerve fibers, will be associated with less of a decrement in speech performance with increasing channel interaction.

Recently, Brochier and colleagues [38] reviewed the aforementioned IPG slope effect in light of possible non-neural factors such as the impedance of stimulating and recording electrodes as well as the electrode-to-modiolus distance (EMD). After analyzing animal and human data and performing calculations with a simple theoretical model, the authors concluded that only the IPG offset effect, stemming from the current difference in dB in a linear portion of the AGF between two IPGs, is independent from any non-neural factors and should be interpreted as an estimate of neural health. Despite this recent finding, the study presented here will also include the logarithmic IPG slope effect to investigate the difference in outcome for both measures and their potential use in future studies.

Also using the telemetry system, the voltage spread from individual electrodes can be assessed by the voltage distribution measured at all electrode contacts in response to stimulation of each individual electrode. This component of the ENI is related to the tissue electrical properties [39] and it reflects the voltage spread in the cochlea. Moreover, it has been suggested that the voltage distribution may reflect the electrode position relative to the inner wall [40, 41] and thus hypothesized that it could explain some variability in speech performance [42]. We hypothesize that CI listeners who have narrow voltage spread across the electrode array will be more resistant in terms of performance decrements in speech understanding when increased levels of channel interaction are applied by increasing number of simultaneously stimulated pairs of electrodes.

The quality of the ENI is also thought to be related to perceptual detection thresholds, especially those obtained with direct focused stimulation [13]. Recently, Bierer et al. [43] introduced a quick and reliable method to measure thresholds across the CI array based on the Békésy tracking procedure [44]. DeVries et al. [16], DeVries & Bierer [15], Kreft et al. [45] as well as Jahn & Arenberg [17] used this method and found significant relationships between focused thresholds and the EMD as well as phoneme identification. The literature suggests that low threshold variability across channels [29, 31] and generally low thresholds [16] are signs of a relatively good ENI, the latter indicating a small EMD. In the present study, the threshold profile was used to characterize the ENI of the participants and explore relationships with speech performance differences between sound coding strategies with increasing amounts of channel interaction. It is hypothesized that the quality of ENI assessed by focused thresholds will impact speech understanding performance differences with sound coding strategies that differ in the amount of channel interaction.

Summarizing, a good ENI might be represented by (I) a large IPG effect, (II) a small, uniform distance between electrodes and the inner wall of the cochlea, (III) low focused thresholds with small variability across electrodes and (IV) a narrow voltage distribution/spread. Channel interactions in the cochlea are heavily influenced by these factors and need to be considered when changing the form of electric stimulation in speech processing strategies for CI users. The individual ENI measures were used to analyze the relationship to speech performance differences with Sequential, Paired and Triplet coding strategies. The main hypothesis is that the amount of decline in speech perception scores with increasing channel interactions (from Sequential to Paired to Triplet) may be explained by the quality of the ENI.

## 2. Methods

### 2.1 Participants

Participants were fourteen postlingually deafened adult CI users (see Table 1). Thirteen were implanted with the HiRes90k system (11 had a Helix, 2 a Mid-Scala electrode array, and one with the Clarion CII implant with a HiFocus 1J electrode array). All CI users had at least six years of experience with their CI and were experienced in performing studies. Participants were offered regular breaks and were free to pause at any time during the experiments. All participants gave written consent prior to the experiment and were paid travel expenses. Ethical consent was granted by the Medical University Hannover.

**Table 1:** Demographic information for all participants of the study.

ID	Age [y]	Gender	Onset Deafness [y]	Duration of Deafness [y]	Experience with CI [y]	Aetiology	Implant
01	61	M	26.9	16.5	11.5	Unknown	Helix
02	68	F	58.3	6.0	9.8	Acute	Helix
03	71	F	61.9	0.1	9.3	Acute	Helix
04	68	F	53.3	0.0	15.3	Genetic	Helix
05	68	F	54.0	0.0	11.9	Genetic	Helix
06	57	M	26.1	7.1	9.3	Ototoxic	Helix
07	64	M	51.3	1.7	11.5	Unknown	Helix
08	23	M	12.0	0.0	6.7	Unknown	Helix
09	24	F	4.0	7.0	12.9	Unknown	Helix
10	64	M	57.0	0.0	6.3	Unknown	Mid-Scala
11	73	M	65.0	2.0	9.3	Genetic	Helix
12	54	M	32.5	0.0	18.0	Acute	CII
13	76	M	58.0	6.1	11.1	Acute	Helix
14	72	M	65.0	1.8	6.2	Acute	Mid-Scala
AVG	60.2		44.6	3.4	10.6		
STD	16.7		20.3	4.7	3.3		

### 2.2 Sound coding strategies

**Fig 1:**
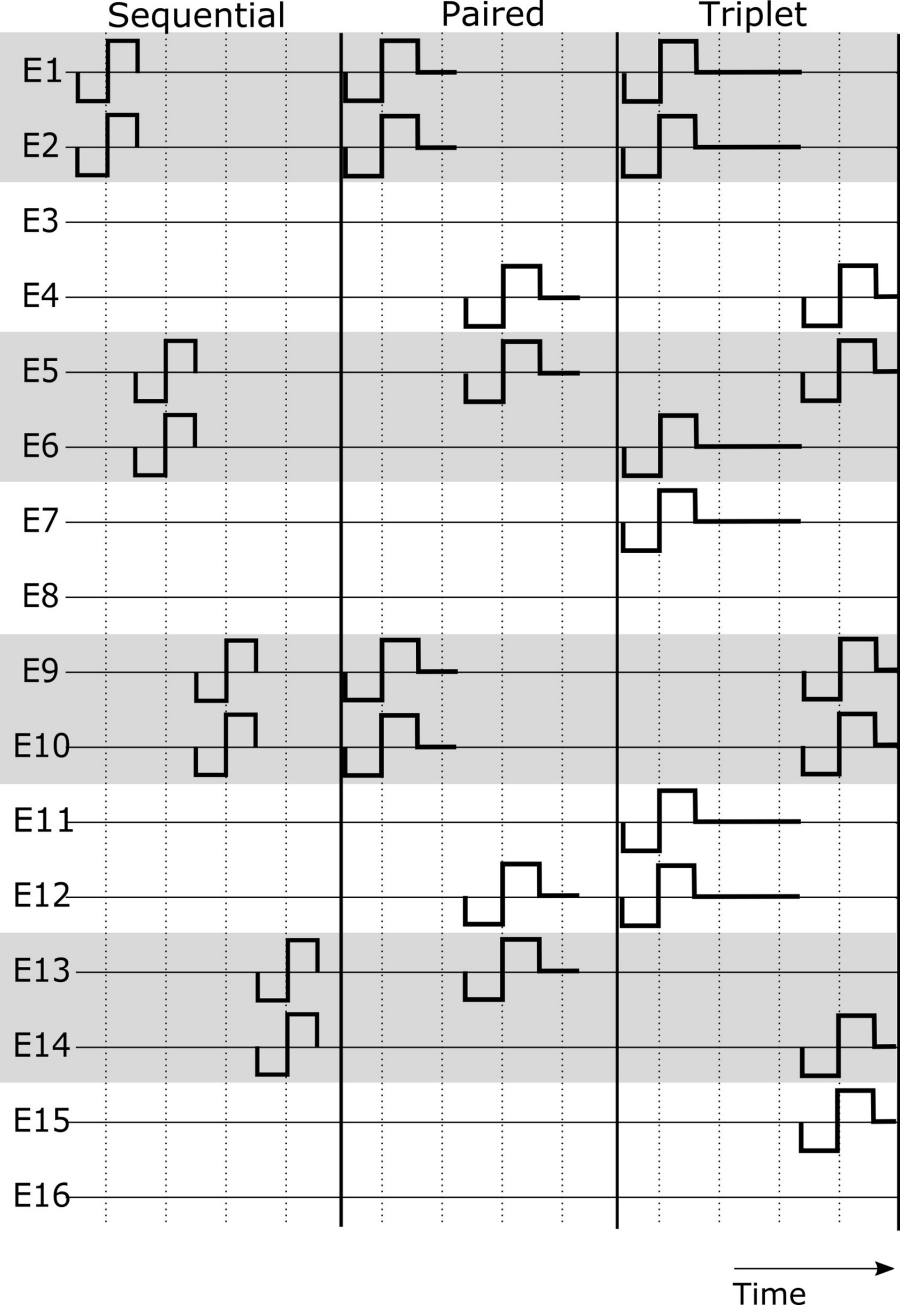
Sequence of stimulation for the sound coding strategies used in the speech performance task. Electrodes over time show the principle of the order of stimulation with one (Sequential), two (Paired) or three (Triplet) channels at the same time.

Speech understanding performance of the participants was tested acutely with three sound coding strategies. The electrode sequence of stimulation is represented in Fig 1. The first strategy was F120 Sequential (named here only Sequential), which is the same strategy that all of the participants used in their everyday settings. It consists of a dynamic range reduction in the form of a front-end automatic gain control, further analyzed with a fast Fourier transformation and a spectral peak locator to adjust the α of the virtual channel between two physical electrodes accordingly [22]. For all strategies a channel consisted of two adjacent electrodes stimulated simultaneously such that the total current is steered between the two electrodes (virtual channel). The second strategy, Paired, simultaneously stimulated two channels that were separated by six electrodes (contrary to the commercial Paired strategy with eight electrode separation) and a zero-phase part at the end of every biphasic pulse was added to maintain the same pulse rate as Sequential. The final strategy is referred to as Triplet, which consisted of simultaneous stimulation of three current steered channels with a fixed distance between channels of three electrodes and an even longer zero-phase included after the end of the biphasic pulse to maintain equal pulse rate. All strategies used the monopolar mode and stimulated with the same pulse duration and pulse rate.

All strategies were loudness balanced according to the procedure described in Langner et al. [27]. First, Sequential was fitted by increasing the current on the first (apical) electrode until the participant stated most comfortable loudness (a 6 on the loudness scaling sheet going from 0 “not heard” to 10 “too load”), repeating this process with all subsequent electrodes. When all electrodes were set to a most comfortable loudness, the strategy was activated and a “Comité Consultatif International Télégraphique et Téléphonique” (CCITT) noise was presented at 65 dB-A SPL free-field via loudspeaker. Globally, the most comfortable levels (MCL, upper limit of stimulation) were changed until the participant perceived the CCITT noise pleasantly as well. For Paired and Triplet, the shape of the fit for Sequential was used but the global MCLs were changed according to the statement of the participant’s perception of the CCITT noise when using the specific strategy. Thresholds were set to 10% of the respective MCL values, corresponding with the manufacturer guidelines and clinical practice at the Medical University Hannover. This procedure is not as accurate as measured thresholds and may reduce low-level speech information that can be beneficial for speech-in-quiet, especially at soft sound levels [3, 46]. In all cases, the current had to be reduced with Paired and Triplet strategies with respect to Sequential. When all three strategies were perceived as pleasantly loud, the following loudness balancing procedure was performed: a strategy was activated and the CCITT presentation started. After a brief time of accommodation for the loudness percept, the strategies were changed while the CCITT noise was still playing. Again, after a brief time with the second strategy, the noise was deactivated and the participant had to state if the second strategy sounded softer or louder than the first one. If a loudness difference was present, the global MCLs were changed accordingly in 2% steps based on the current MCL for the second strategy. The order of the loudness balancing was Sequential vs. Paired, Paired vs. Triplet and Triplet vs. Sequential. The latter balancing was only performed to check if the loudness balancing was performed correctly between all strategies. The Sequential strategy was always the starting point that was not altered. This procedure was performed until the participant stated no perceivable loudness difference between all three strategies. The MCL differences in dB between each pair of strategies (Sequential and Paired, Paired and Triplet, Sequential and Triplet) was used as an estimation of electric interactions when adding additional simultaneous stimulating channels.

### 2.3 Speech understanding performance

Speech understanding performance was assessed with the Hochmair-Schulz-Moser (HSM) sentence test [47]. The test contains day-to-day sentences, including questions, of various lengths and topics and is organized in lists of 20 sentences. Each condition (sound coding strategies, see section 2.2) was tested with one training list and two test lists, each of which was randomly chosen from the 30 given lists. The test was performed free-field at a sound pressure level of 65 dB SPL-A (with the speech fixed at that level) with the CCITT used as calibration signal. This CCITT signal was also used as the noise to create the individual signal-to-noise ratios (SNR) during the measurement. The participant’s task was to repeat everything he/she understood, even if only words or parts of a word were understood. The output of each list was the number of correctly repeated words in percent. Each participant ran through a number of trial-and-error runs with the speech material being presented at different SNRs with the Sequential strategy to identify the SNR at which the participant understood roughly 50% of the speech material. This SNR was then used throughout the testing with all strategies. The order of the strategies tested was randomized across participants, with a total of 6 possible orders distributed among them. The participant was blinded to the strategy being tested. If the difference in percent correct between the two test lists was greater than 20%, a third test list was used for additional testing and the one deviating excluded from averaging.

Additionally, speech understanding performance data from the clinical routine was gathered. Those data were measured with the personal sound processor and fitting of the F120 Sequential strategy by an audiologist. One list of the HSM sentence test was used in a quiet setting and one list was used with an SNR of +10 dB. The performance in quiet with monosyllables was assessed with the “Freiburger Einsilber” monosyllabic test material with one list as well [48]. Everything was measured in free-field at 65 dB-A SPL. The data were gathered from the patient database of the German Hearing Center Hanover and were comprised of the average of the last two appointments not older than two years from the time of the study.

### 2.4 Voltage spread

Intracochlear current spread or voltage spread was measured with the Electrical Field Imaging and Modeling Tool [EFIM, Advanced Bionics, Valencia, USA, see 33] by stimulating each electrode in turn in monopolar mode and measuring the potential at each of the other fifteen electrodes consecutively. Participants used a Platinum Sound Processor linked to a Clinical Programming Interface connected to a standard PC. Software used for stimulation was the research software BEDCS (Bionic Ear Data Collection System, version 1.18.377, Advanced Bionics). Sub-threshold monopolar stimulation was used with a biphasic pulse with an amplitude of 40 μA and pulse duration of 66.45 μs. Recordings from all electrodes across the array–stimulating and recording–were collected. Results from the EFI recording were constructed by omitting the recorded potential at the stimulating electrode and fitting an exponential curve based on the data of the fifteen recording electrodes. The resulting peak of the exponential fit was used to determine the width of the curve 3 dB below this point in mm [39, 40, 42]. Most extreme electrodes were created by mirroring the side towards the middle of the array. The two most apical and basal electrodes (1, 2, 15 and 16) were excluded from the analysis, because not enough data were available to fit an exponential function beyond these electrodes.

### 2.5 Focused thresholds

The method of measuring hearing thresholds with focused stimulation stems from the work of Bierer and colleagues [43] and was used in a few recent studies [14–16, 43]. It follows the idea of the Békésy tracking, in which a tone is constantly presented and its frequency shifted slowly from low to high [49]. In the case of CI users, biphasic current-steered pulse trains were used and the peak of the voltage spread was slowly shifted from the basal to the apical region of the cochlea, mimicking the frequency shift from low to high frequencies. More details can be found in Bierer et al. [43].

The software and procedures used by Bierer and colleagues [43] were implemented in the present study (Matlab, Mathworks, Boston, USA) which interfaced with the research software BEDCS (Version 1.18.317). Briefly, stimuli were biphasic, charge-balanced, cathodic-first pulse trains with phase durations of 97.2 μs and a pulse rate of 997.9 pulses per second (pps). Pulse trains had a duration of 200.4 ms and a silence period of 300 ms to result in a repetition period of roughly 500 ms and were presented in the focused, current steered quadrupolar (QP) mode. The level of stimulation changed in 1-dB steps depending on the listener’s response and the steering coefficient was swept across electrodes with two presentations of each coefficient.

**Fig 2:**
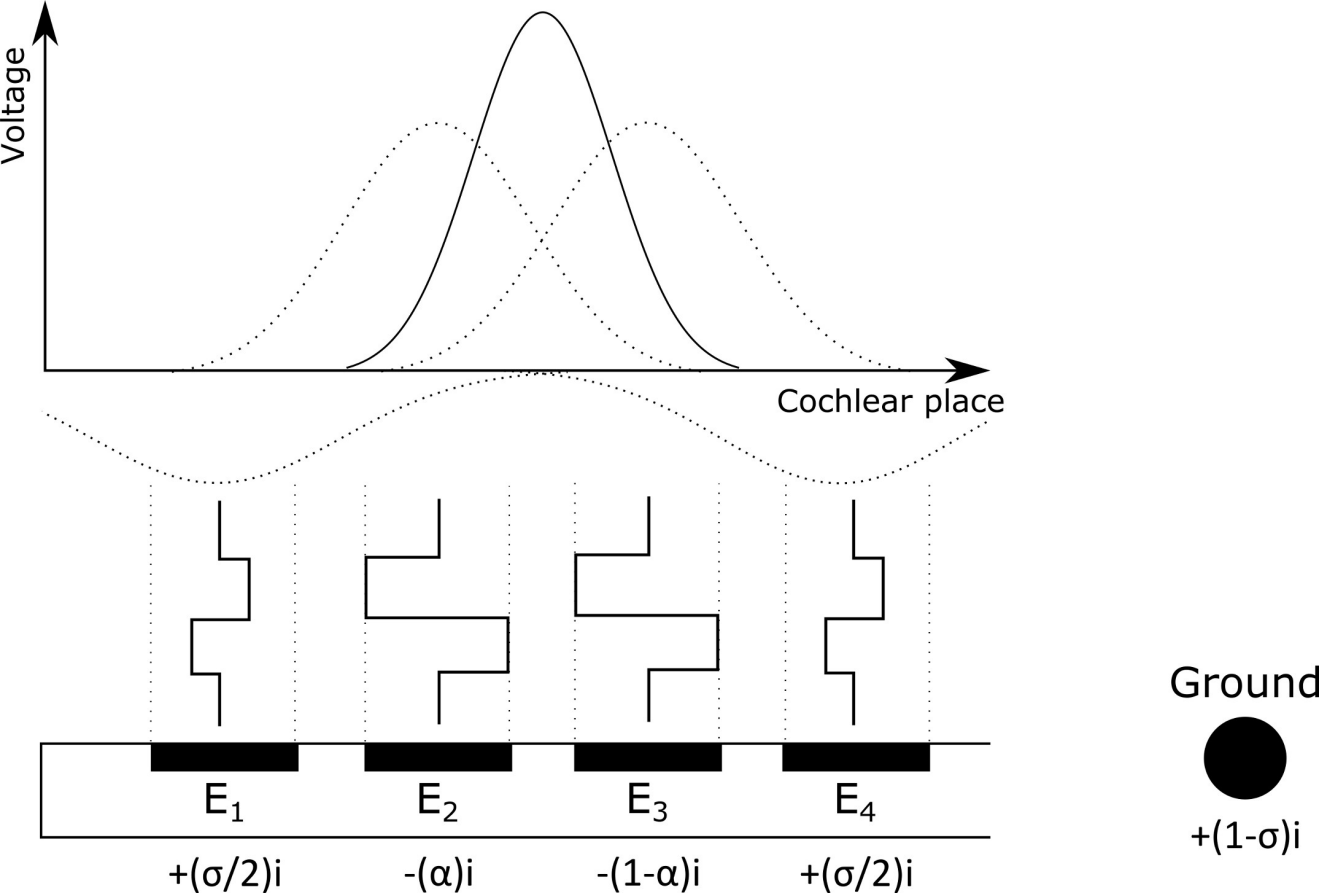
Quadrupolar stimulation mode: Two electrodes (E_2_ and E_3_) stimulate with the current divided by α with cathodic-first biphasic pulses. The other adjacent electrodes E_1_ and E_4_ act as anodic-first return electrodes in which the current is divided by σ between the two electrodes and the ground electrode. The effective current spread across cochlear place is depicted above as a solid black line, while the individual current spread of each electrode is depicted as dotted lines.

Quadrupolar (QP) mode was used to focus the electrical fields, and simultaneously stimulates four adjacent electrodes (see Fig 2): the center two electrodes (E_2_ and E_3_) with cathodic leading biphasic pulses use current steering, while the other two, flanking electrodes (E_1_ and E_4_) act as return electrodes by using anodic leading biphasic pulses. For this experiment, a focusing coefficient, σ, of 0.9 was chosen to deliver most of the return current (0.9 out of 1) to the adjacent, active electrodes and 0.1 to the ground electrode. This leads to focusing the current spread across the cochlear place substantially when compared to a stimulation in monopolar mode (i.e., without electrodes E_1_ and E_4_ when all of the return current is delivered to the ground electrode. For more details regarding the stimulation mode see Bierer et al. [43].

The sweep thresholds were measured once for an upward sweep (from apex to base) directly followed by a downward sweep (base to apex). The α factor was altered in 0.1 steps to sweep through the electrodes. Due to the location of the return electrodes in the QP mode, stimulation of the first and last (sixteenth) electrode was not possible in the sweep. The sweep procedure started at 3 dB below MCL. If the spacebar was pressed, the current level was decreased by 1 dB and if it was released, it was increased by the same amount. The threshold estimates were calculated with a weighted averaging of current levels alongside both sweeps. For a detailed description see Bierer et al. [43]. The results were originally in μA stimulating current and were converted to dB rel. 1 μA. Note that the QP mode was used only to assess the quality of the electrode-neuron interface and was therefore different to the monopolar virtual channels that were used in the sound coding strategies to assess speech understanding performance.

### 2.6 Amplitude growth functions

eCAPs were obtained with a forward-masking paradigm [50, 51] with the BEDCS software (version 1.18.337). Stimuli were monopolar, biphasic, cathodic first pulses with 32 μs phase duration. The IPG was 2.1 μs or 30 μs for both masker and probe, and the masker probe interval (from onset to onset) was set to 400 μs. Every odd electrode number was used as stimulating electrode, while the recording electrode was two electrodes basal from the stimulating one (except for electrode 15, for which electrode 13 was used for recording). While recording, the internal amplifier was set to a gain of 300, the sampling rate was 56 kHz and 32 trials were recorded for averaging. Stimulating current levels varied across participants and were increased consecutively until the participants stated that the stimulus was perceived as loud (equal to an 8 on a loudness scaling sheet ranging from 0 “no sound” to 10 “too loud”). Averaged eCAP waveforms were displayed to the examiner for a visual inspection to ascertain if a response was present. The eCAP amplitude was calculated as the difference in amplitude (mV) between the N1 (first negative peak at around 0.2–0.4 ms) and P1 (later positive peak at 0.6–0.8 ms after the probe onset). Amplitudes below 0.03 mV were discarded and considered as “no response”. AGFs were created by plotting the eCAP amplitudes as a function of stimulation current. A linear and sigmoidal fit was applied to the AGFs. A sigmoidal fit was only applied when at least 5 data points (eCAP amplitudes) were successfully recorded. Their slopes and peak amplitudes were analyzed, similar to Ramekers et al. [34]. As suggested by Brochier et al. [38] we use the level 50%, which is the current difference in dB at the 50% point of the sigmoidal fit, as the linear portion of the AGF. Since it is assumed that ENI factors such as impedance influence the IPG slope effect across electrodes [52], the statistical evaluation of said effects was only performed when sufficient eCAP data (robust sigmoidal fit with at least 5 data points) was present for both IPG conditions. Recordings for some of the participants only contained one or two recording electrodes that fulfilled this requirement, and the statistical analysis was performed for the within individual average of all individual pairs and compared across participants. For the investigation of a linear relationship with ENI measures and speech understanding performance differences, the averaged data across electrodes for each participant was used. The maximum stimulation current amplitude at which the participant stopped the eCAP measurement was also used as an analysis parameter.

### 2.7 Statistical analysis

For participants ID02 and ID07 it was not possible to obtain eCAP responses and therefore they were excluded from specific statistical analyses that used eCAP data. SPSS statistical software was used for the analysis of the data (IBM Corp. Released 2017. IBM SPSS Statistics for Windows, Version 25.0. Armonk, NY: IBM Corp.). Simple linear regression models were used to analyze the data and to answer the main hypotheses: can subjective and objective measures describe the speech understanding performance differences in CI users produced by increasing the amount of channel interaction with different sound coding strategies? Additional relationships between demographics and the measured data were analyzed as well. If not stated otherwise, the linear regressions were calculated with the averaged values across measurements/electrodes (see Tables 2 and 3).

**Table 2:** Average (first value) and standard deviation (in brackets) across electrodes for Focused Thresholds (FT), slope of the AGF for IPG = 2.1 μs (AGF_S2_) and 30 μs (AGF_S30_), difference in slope between both IPGs (IPG Effect), the IPG offset effect, the MCL difference between Sequential and Paired (SP) and Sequential and Triplet (ST) as well as the 3 dB width below the peak of the exponential fit of the EFI recording (EFIW).

ID	FT [dB]	AGF_S2_ [mV / dB rel. 1 μA]	AGF_S30_ [mV / dB rel. 1 μA]	IPG Effect [mV/ dB rel. 1μA]	IPG Offset [mV / dB rel. 1μA]	MCLDiff_SP_ [dB]	MCLDiff_ST_ [dB]	EFIW [mm]
**01**	43.2	0.032				0.36	1.88	1.19
**02**	47.0	0.114	0.107	0.006	2.19	2.60	5.15	1.16
**03**	37.3					1.98	3.38	1.84
**04**	43.0	0.060	0.045	0.001	1.98	1.04	2.54	1.41
**05**	25.1	0.024	0.027	-0.004	2.27	0.84	1.33	1.02
**06**	43.6	0.062	0.061	-0.004	1.46	-0.75	2.42	1.04
**07**	40.1					1.69	2.40	1.64
**08**	47.6	0.078	0.113	-0.012	3.97	2.19	3.41	1.41
**09**	40.3	0.082	0.114	-0.023	1.29	1.21	2.73	2.20
**10**	45.6	0.053	0.059	-0.005	1.54	2.51	4.41	1.77
**11**	40.7					0.86	2.21	1.16
**12**	41.7	0.075	0.080	0.005	1.70	1.61	3.16	1.19
**13**	41.8	0.027	0.060	-0.034		0.57	1.79	1.49
**14**	44.7	0.059	0.067	-0.006	-1.71	2.92	4.47	1.58
**Summary**	41.6 (5.3)	0.061 (0.027)	0.073 (0.029)	-0.010 (0.017)	-1.54 (2.59)	1.40 (1.01)	2.95 (1.12)	1.43 (1.10)

**Table 3:** Averaged speech understanding performance results in percent correct at individual SNRs for 50% for Sequential (SI_Seq_) and the resulting performance for Paired (SI_Paired_) and Triplet (SI_Triplet_). The difference in performance between Sequential and Paired (ΔSI_SP_) and Sequential and Triplet (ΔSI_ST_) are shown as well as the results from clinical routine with the participants’ speech processor for quiet (CSI_Quiet_) and noise at +10 dB SNR (CSI_Noise_) from the last two appointments (not older than two years). Monosyllables are also collected from the clinical data (CSI_Mono_).

ID	SNR [dB]	SI_Seq_ [%]	SI_Paired_ [%]	SI_Triplet_ [%]	ΔSI_P_ [%]	ΔSI_T_ [%]	CSI_Quiet_ [%]	CSI_Noise_ [%]	CSI_Mono_ [%]
**01**	3	57.0	71.5	73.5	-14.5	-16.5	99.5	68.0	82.5
**02**	5	60.5	43.5	31.5	17.0	29.0	95.0	39.0	72.5
**03**	4	76.0	75.0	59.0	-1.5	17.0	98.0	72.0	70.0
**04**	3	39.0	38.5	63.0	0.5	-24.0	100	92.0	85.0
**05**	3	70.0	71.5	62.0	-1.5	8.0	75.5	40.0	62.5
**06**	1	81.0	75.0	53.5	6.0	27.5	90.0	83.0	95.0
**07**	11	58.5	32.0	16.0	26.5	42.5	100	56.6	75.0
**08**	0	62.0	66.0	67.0	-4.0	-5.0	100	83.5	95.0
**09**	10	58.0	60.0	59.0	-2.0	-1.0	100	57.5	72.5
**10**	3	59.5	68.5	70.0	-9.0	-10.5	100	74.0	80.0
**11**	7	61.5	60.5	53.0	1.0	8.5	96.5	47.5	55.0
**12**	2	65.5	43.0	44.5	22.5	21.0	100	79.0	80.0
**13**	7	54.0	40.0	24.0	14.0	30.0	89.5	25.0	52.5
**14**	-2	58.0	46.5	44.5	11.5	13.5	98.0	62.5	52.5
**Summary**	**4 (3.6)**	**61.4 (9.5)**	**56.5 (14.7)**	**49.9 (17.7)**	**4.8 (11.5)**	**10.6 (20.8)**	**95.9 (6.9)**	**62.8 (19.6)**	**73.5 (14.1)**

## 3 Results

The averaged results from all participants are shown in Table 2.

### 3.1 Speech understanding performance

**Fig 3:**
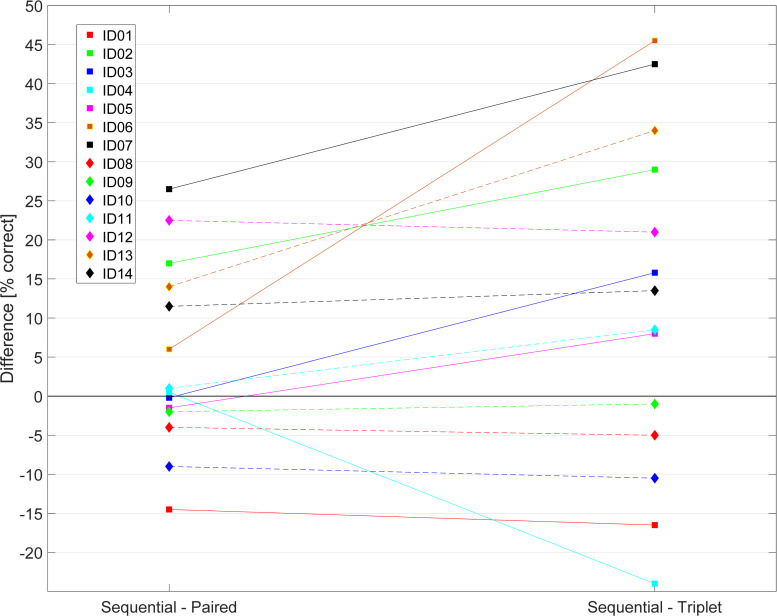
Speech understanding performance differences from all participants between Sequential and Paired as well as Sequential and Triplet.

Table 3 shows the SNR used to reach around 50% speech understanding performance with the Sequential strategy, the resulting performance for all three sound coding strategies at that SNR, the difference between Sequential and the other experimental strategies, as well as the clinically-obtained speech performance data (labeled CSI). The average unsigned difference between the two test lists is 7.76%. A simple linear regression was calculated to test if the two runs correlated with each other to assess the test-retest reliability. The speech understanding performance achieved in the first run/list did explain the performance achieved in the second run/list, F(1,40) = 89.42, p < .001, r² = .691. Furthermore, another simple linear regression was performed to check if the speech understanding performance differences differed between both runs/lists. No significant correlation was found for Sequential and Paired, F(1,12) = 3.532, p = .084, r² = .227. However, the difference in the first run could explain the difference of the second run for the Sequential and Triplet condition, F(1,12) = 29.809, p < .001, r² = .713. On average, the Sequential strategy (SI_Seq_) results in higher speech performance than Paired (SI_Paired_) which is higher than Triplet (SI_Triplet_). Overall differences between Sequential and Paired (ΔSI_P_) are 5% with a standard deviation of 12%. The difference between Sequential and Triplet (ΔSI_T_) is larger with 11% on average and a standard deviation of 21%. Fig 3 displays the individual speech understanding performance differences. While some participants decrease in performance when increasing the number of simultaneously stimulating channels (ID02, 03, 07, 12, 13 and 14), others increase in performance (ID01, 08 and 10). Some participants only decrease in performance with the Triplet while reaching the same performance with Sequential and Paired (ID05, 06, 11). Participant ID04 showed similar performance with Sequential and Paired and an increase when using Triplet. The performance of Participant ID09 stayed similar for all strategies. A repeated measures of ANOVA (rmANOVA) with Greenhouse-Geisser correction applied was performed to investigate an effect of the strategy on speech performance. A significant effect was found (F(1.324, 17.206) = 3.416, p = .048). Post hoc tests, however, revealed no significant differences between strategies when the Bonferroni correction for multiple testing was applied. Only a trend between performance differences Sequential and Triplet were found with p = .067. Another rmANOVA with sphericity assumed was performed to check for an effect of strategy on the MCLs. There was a significant effect of strategy, F(2,26) = 39.936, p < .001. Post-hoc Wilcoxon signed rank tests with Bonferroni correction revealed significant differences between the MCLs of Sequential and Paired (average of 1.40 dB; Z = 2.919, p = .011), Sequential and Triplet (average of 2.95 dB; Z = 3.296, p = .003) as well as Paired and Triplet (average of 1.55 dB; Z = 3.296, p = .003).

For the linear regression models on the individual data, the relative differences between Sequential and Paired (ΔSI_P_) and Sequential and Triplet (ΔSI_T_) as shown in Fig 3 were used. The following sections investigate our hypothesis that the ENI measures (voltage spread, focused thresholds and AGF characteristics) may explain these relative speech performance differences. Overall, no demographic measure or the absolute speech scores for each strategy was able to explain these differences.

### 3.2 Voltage spread

**Fig 4:**
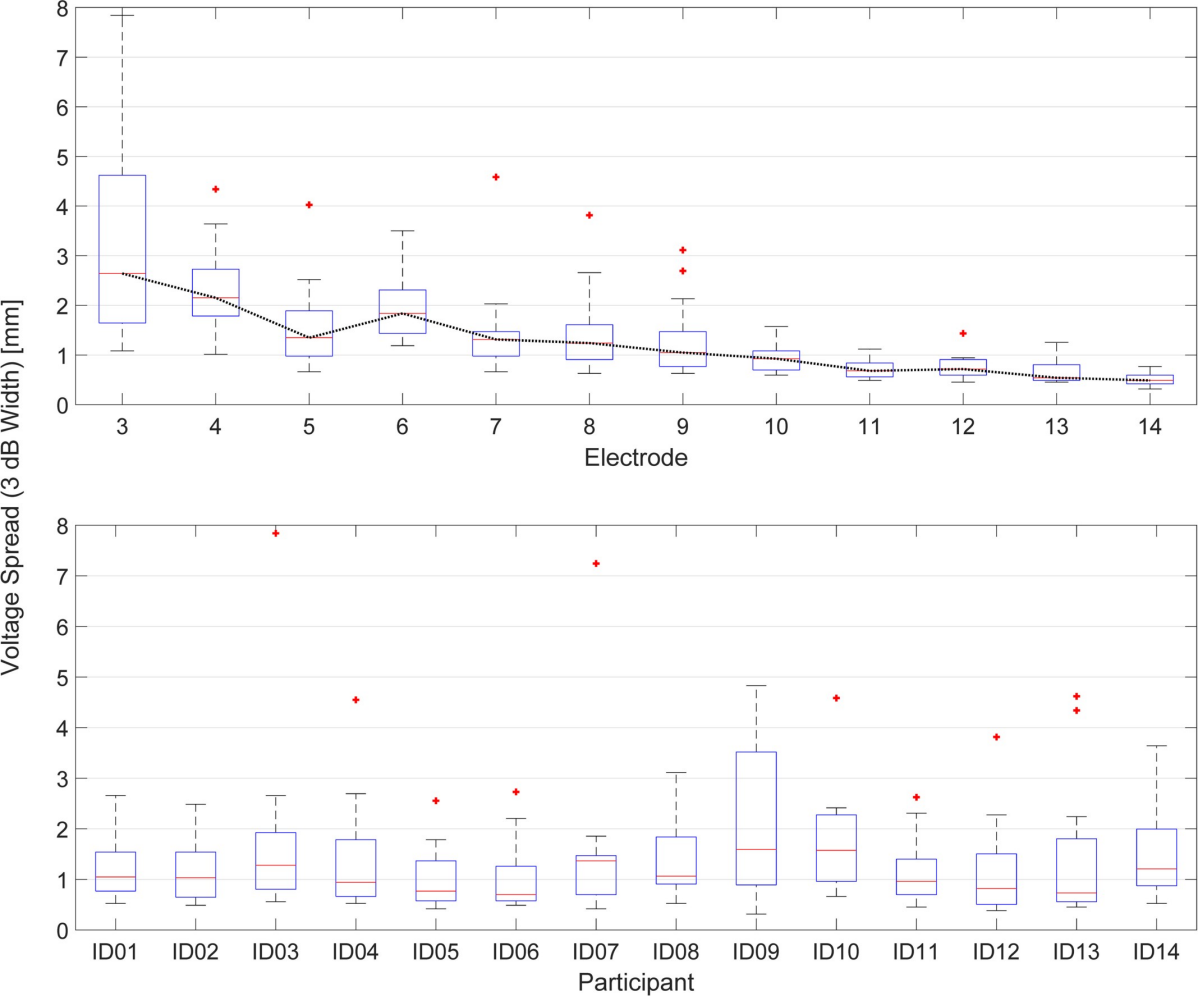
EFIW as a function of electrode number averaged across participants (top plot) and as a function of participants averaged across electrodes (bottom plot). The median is in red, edges show the 25th and 75th percentiles and whiskers the most extreme data.

The results from the EFI measurement in the form of EFIW are listed in Table 2 and displayed as a function of electrode number (across participants) and as a function of participants (across electrodes) in Fig 4. The average EFIWs range from 1.08 to 2.46 mm (M = 1.59 mm, STD = 1.19). An rmANOVA with Greenhouse-Geisser correction applied was performed to investigate an effect of the recording electrode on the EFIW. A significant effect of the stimulating electrode was found to show that EFIW decreased from apex to base (F(3.458, 38.041) = 9.957, p < .001). Descriptively, apical electrodes show broader voltage spread than basal ones. There is a continuing decrease in EFIW going from electrode 3 to 14. Similar voltage spread distributions can be seen across participants, except participant ID09, who showed a broader distribution as well as a higher median and 75% percentile across electrodes. The EFIW did not correlate with speech performance differences (F(1,10) = 0.012, p = 0.914 for ΔSIP and F(1,10) = 0.013, p = 0.910 for ΔSIT).

### 3.3 Focused thresholds

A simple linear regression was calculated to test if the two runs correlated with each other to assess test-retest reliability. The change across electrodes in the first run did explain the change across electrodes in the second run, F(1,192) = 1742.7, p < .001, r² = .885. FTs range from 25.1 to 47 dB rel. 1 μA (M = 41.6 dB rel. 1 μA, STD = 5.3). One participant, namely ID05, exhibited fairly low thresholds on average 25.1 dB rel. 1 μA compared to the group average of 41.6 dB rel. 1 μA. The rmANOVA revealed, that focused thresholds did not correlate significantly with the relative speech performance differences between Sequential and Paired ΔSI_P_ (F(1,10) = 2.408, p = 0.151) or Sequential and Triplet ΔSI_T_ (F(1,10) = 2.820, p = 0.124). However, the MCL difference of Sequential and Triplet positively correlated with the average focused thresholds (F(1,12) = 6.897, p = .044, r² = .364) and their standard deviation (F(1,12) = 6.884, p = .044, r² = .364).

### 3.4 Amplitude growth functions

**Fig 5:**
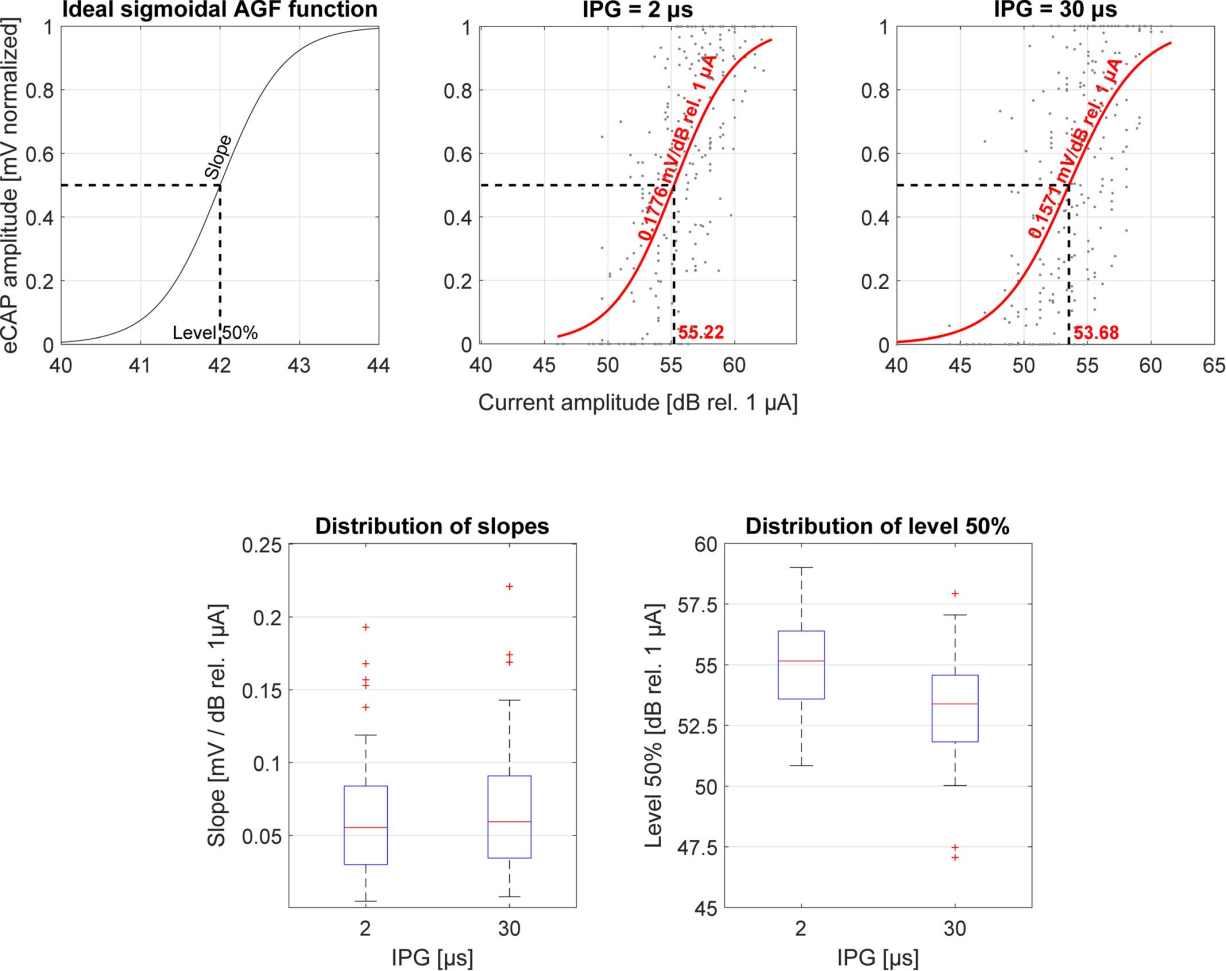
Ideal AGF derived from a sigmoidal fit (upper left plot) and assessed AGFs across all participants fir IPG = 2 μs (upper middle plot) and 30 μs (upper right plot). Distribution of slopes (bottom left plot) and level 50% to calculate the significant dB offset effect (bottom right plot).

The average slopes of the AGF generated from the eCAP measurements with IPG = 2.1 μs (AGF_S2_) and with 30 μs (AGF_S30_) are listed in Table 2 for all participants. Fig 5 shows the distribution of the AGF slopes across participants as a function of the recorded electrode. An rmANOVA with Bonferroni correction applied was performed to test the hypothesis that there is an IPG slope effect: a significant difference between the slopes of the AGFs of each IPG setting. There was no such effect on the AGF slopes in logarithmic units, F(1,41) = 0.399, p = .530. The level 50% points showed a significant difference between IPG conditions (F(1,30) = 10.617, p = .002), suggesting an IPG offset effect (see Fig 5). Since no IPG effect was found for the logarithmic AGF slopes, only the IPG offset effect was used as an ENI measure for correlation with the speech understanding performance differences. No statistically significant relationship between IPG offset effect and Sequential/Paired (F(1,8) = .0877, p = .376), Sequential/Triplet differences (F(1,8) = 1.027, p = .340) was found.

## 4. Discussion

This study investigated the potential contributions of behavioral (focused thresholds) and objective measures (voltage spread and eCAP characteristics) to explain the change in speech perception scores as a result of increased channel interactions from adding simultaneously stimulated channels to speech processing strategies. Consistent with previous studies, the clinical speech perception results correlated significantly with the average focused thresholds and with threshold variability, which in turn were positively correlated with the MCL difference between Sequential and Triplet. The logarithmic IPG slope and offset effect were not able to explain the individual changes in performance with the number of simultaneously stimulated channels in a strategy, although a statically significant IPG offset effect was observed in contrast to the logarithmic IPG slope effect.

### 4.1 Speech understanding performance

The speech understanding performance results from all three sound coding strategies followed a rough pattern, in that increasing the number of simultaneous stimulating channels reduced the current necessary for the same perceived loudness. This behavior was also reported in Langner et al. [27]. Although a significant effect of strategy was found in the rmANOVA, no significant difference in speech performance was found between Sequential and Paired and Sequential and Triplet in the post-hoc tests, although a trend was seen for the Sequential-Triplet difference. This is in partial agreement with the earlier study by Langner et al. [27] who found a significant difference between Sequential and Triplet, with Sequential resulting in significantly higher speech intelligibility. It is important to state that the performance with Paired and Triplet was assessed acutely; therefore, improvements by habituation with both strategies are probable. The performance differences (ΔSI_P_ and ΔSI_T_) show various outcomes also reflected in the earlier study, which were described in the introduction. As mentioned before, using behaviorally measured thresholds would have added robustness to the speech understanding performance data and probably improved performance as well [3, 53]. Another way to verify the fit of the strategies is the measurement of sound field thresholds [3], which should have been incorporated into the methods as well to increase robustness.

Near-field interactions of sequentially stimulated channels need to be considered when stimulation takes place in quick succession. Similar to simultaneous stimulation, electric interaction during sequential stimulation is greatest when the electrodes are adjacent and decrease with larger separation [54]. Comparing the three strategies investigated here, adjacent channels are stimulated sequentially sooner with Triplet and Paired compared to Sequential (for the most extreme case, see electrodes 6 and 7 directly followed by electrodes 4 and 5 with Triplet). These tempo-spatial interactions might have an influence on the loudness percept as well as on the speech performance understanding and should be considered when investigating simultaneous sound coding strategies. Spatial interactions from simultaneous stimulation have been investigated by Langner et al. [28], who found a linear loudness decrease of 0.24 dB / mm electrode spacing with a maximum of 4.52 dB current amplitude difference for adjacent channels. This is also reflected in the significant effect of strategy on MCLs shown in this study, in which Triplet with the smallest distance between channels shows the lowest MCL with respect to the other strategies. Temporal interactions can for example be investigated with the effect of the inter-pulse interval on loudness, which was done by McKay et al. [55]. The authors found between 1- and 2-dB current reduction necessary for equal loudness between two-pulse per period and single-pulse/period stimuli, independent of inter-pulse interval. From these previous studies, we assume that the simultaneous presentation of virtual channels in this particular setup creates far more spatial interaction than temporal interaction.

A surprising finding is the lack of a significant relationship between all the ENI measures and the time of experience with the CI. The latter is a presumed predictor for speech performance [56, 57], although a recent study suggested the contrary [58]. One might assume that both variables would correlate, however, all of our study participants had long durations of CI use (6 years and longer). A larger sample of participants, some of whom had shorter durations of implant use may have changed this finding.

### 4.2 Voltage spread

The constant decrease in EFI width from apex to base is consistent with the findings of Vanpoucke et al. [59], who found increasing voltage drops when going from apex to base, effectively reducing the width of the EFIW as computed in our study. The average voltage spread (EFIW) was not able to explain the speech understanding performance differences.

### 4.3 Focused thresholds

The linear regression analysis revealed that the test-retest reliability of the focused threshold method was very good. The fairly low focused thresholds of participant ID05 were achieved with a Helix implant, a modiolus-hugging electrode array. While the electrode array is an indicator for lower thresholds compared to the Mid-Scala or 1j, other participants with the same array did not have such low thresholds. When the data of ID05 is handled as an outlier, no change on the relationships to other measures occurred.

The MCL difference between the strategies can be seen as a direct measure of electric interaction and loudness and correlated positively with the average and standard deviation of the focused thresholds. This means that a higher MCL difference (higher current amplitudes across channels for Sequential) corresponds to higher behavioral thresholds and a larger standard deviation. Both of these aspects are thought to represent a poor ENI, indicating a region with low neuron survival or poor electrode placement [12–14, 29]. This relationship suggests that CI users with a poor ENI experience much stronger electric interactions with simultaneous stimulation which necessitates a stronger reduction in current amplitude to reach a comfortable loudness.

### 4.4 eCAP measures and amplitude growth functions

Because the first phase of the biphasic pulse introduces charge into the cochlea, a longer IPG results in a longer lasting charge and therefore increased neural excitation and an increased firing rate. It therefore seems logical that the slope of the AGF would be steeper for the IPG = 30 μs than for the 2 μs condition. However, we did not find a significant difference between the logarithmic IPG slopes, with only a 0.205 mV / dB rel. 1 μA difference. As mentioned before, changes experienced by neural structures when performing different stimulations are best represented on the logarithmic scale [60]. Brochier et al. [52, 61] argued that using a linear abscissa and ordinate leaves the slope measurement susceptible to non-neural factors such as impedance and EMD and that the IPG offset effect would negate these factors. While we observed a significant linear IPG slope effect, the logarithmic scaling reduced the difference in slope so that significance was lost. The IPG offset effect was usable as an ENI correlate in that the difference between the 50% points of the sigmoidal fit (the mentioned linear portion) between both IPG conditions was significant. However, none of the AGF characteristics were able to explain the speech understanding performance differences or relate to any other ENI measure such as focused thresholds or voltage spread. This suggests that focused thresholds and voltage spread relate to different aspects of neural health or the ENI than the IPG slope and offset effect. Furthermore, it suggests that performing correlation analyses between averaged measures (especially for ENI measures that can change drastically across electrodes) is not robust.

Schvartz-Leyzac & Pfingst [37] found a relationship between the linear IPG slope effect and the CUNY sentences as well as consonants in quiet. More specifically, they found a significant correlation between the individual ear/device differences in IPG effect and speech understanding performance and concluded that a higher ear difference in IPG effect explained a lower/negative ear difference in speech reception threshold, effectively telling which ear/device performs better. By analyzing each participant across both ears, a significant correlation was possible. Inspired by the study of Schvartz-Leyzac & Pfingst [37] it was predicted that a significant relationship between speech understanding performance differences and the linear IPG slope effect would be observed in the present study. However, no significant linear IPG slope effect was found. This might stem from difference data assessed with a measure of high variability (speech-in-noise) or the more exact method of acquiring the speech reception thresholds as used by Schvartz-Leyzac & Pfingt. Brochier et al. [38] argued that an averaging of the IPG slope effect across electrodes would reduce the validity of the measure due to an averaging of neural health across the cochlea.

Further investigations are needed to design measures for more detailed characterization of the electrode interface in CIs, especially in a clinical environment. Moreover, it is possible that the inclusion of measures that account for cognitive abilities improves the prediction of speech understanding performance differences with different sound coding strategies. Imaging data, detailing the placement of the electrode array in the cochlea, can help to improve the characterization of the individual users’ ENI. It has been shown that the electrode-to-modiolus distance correlates with focused threshold, but not with speech performance [16, 17]. Using imaging data might have improved the current data set with regards to the assessed focused thresholds, but it is unlikely that it would have served as a predictor of the speech understanding performance difference when the average and variability of focused thresholds did not. A larger sample of participants is also needed to increase statistical power and possibly allow for the categorization of participants into groups for whom performance was unaffected or improved by increasing the number of simultaneously stimulated pairs of electrodes, and those whose performance declined.

## 5 Conclusions

The estimated quality of the electrode-nerve interface was measured in listeners who also participated in a speech perception experiment with various stimulation strategies that varied in the degree of channel interaction. A trend towards worse speech understanding performance with increasing the number of simultaneously stimulated channels was observed; however, the planned comparisons were not significant after type II error correction, probably caused by large inter-subject variability. Impedance and trans impedance measures show that apical electrodes present broader voltage spread than basal ones. Two measures of neural health were investigated, the IPG slope/offset effect of AGFs and focused thresholds. The IPG offset effect reached significance in that the 50%-point of the sigmoidal fit differed between IPG conditions, but the logarithmic IPG slope effect failed to do so. The IPG offset effect did not result in a significant correlation with differences in speech scores. Focused thresholds did not correlate significantly with the relative speech understanding performance differences, but significantly correlated with the MCL difference between Sequential and Triplet. This relationship indicates that a poor ENI corresponds to stronger electric interactions with simultaneous stimulation, requiring greater reductions in current amplitude for comfortable loudness.

## Supporting information

S1 Data (XLS)
